# Autologous Stem Cell Transplantation in Multiple Myeloma with Renal Failure: Friend or Foe?

**DOI:** 10.1155/2019/9401717

**Published:** 2019-10-29

**Authors:** Hongfei Zhong, Xiaojie Xie, Gaosi Xu

**Affiliations:** ^1^Department of Nephrology, The Second Affiliated Hospital of Nanchang University, Jiangxi, China; ^2^Grade 2015, The Second Clinical Medical College of Nanchang University, Jiangxi, China; ^3^Department of Nephrology, 908 Hospital of People's Liberation Army, Yingtan, China

## Abstract

Autologous stem cell transplantation (ASCT) is a standard treatment for multiple myeloma (MM), but the clinical response and renal curative effect in MM patients with renal failure (RF) remain controversial. The myeloma kidney disease has different types, and most are due to the direct toxic effects of light chain. Although ASCT can effectively clear the light chain, the data of renal function improvement are still limited. We reviewed the published literatures, focusing on the prospective studies, the retrospective analysis studies, and the case reports. RF patients who received ASCT displayed a low survival rate (OS: HR 1.95, 95% CI 1.020 to 3.720; *I*^2^ = 64.9%, *P* = 0.014) and a shorter EFS/PFS (EFS/PFS: HR 1.53, 95% CI 1.090 to 2.140; *I*^2^ = 0%, *P* = 0.669). However, ASCT was feasible and could have the similar clinical response outcomes compared with the normal renal function (CR: OR 1.013, 95% CI 0.569 to 1.804; *I*^2^ = 48.5%, *P* = 0.101; PR: OR 1.013, 95% CI 0.342 to 1.226; *I*^2^ = 46.3%, *P* = 0.144). Moreover, MM with RF after ASCT had a good improvement of renal function and melphalan is still an important factor affecting the treatment of ASCT.

## 1. Introduction

Renal failure (RF) is one of the most common complications of multiple myeloma (MM), and it has been associated with higher risk of mortality and increased hospitalization rates due to complications such as electrolyte abnormalities, catheter-related complications, and infections [1, 2]. Factors contributing to myeloma kidney disease include hypercalcemia, dehydration, hyperuricemia, amyloid deposition and plasma cell infiltration, light chain-induced proximal tubular damage, cast nephropathy, and interstitial nephritis [3]. Furthermore, administration of nephrotoxic medication, dehydration, and hypercalcemia always adds to the development of acute kidney injury (AKI) [4–6]. Improved renal function is an important therapeutic aim and has become a predictor of better outcome in MM [7].

Autologous stem cell transplantation (ASCT) as a standard treatment for MM because of its association with longer event-free survival (EFS) and higher complete response (CR) rate [8, 9], it has been the mainstay of therapy in young patients (age < 65 years) with MM [10]. Historically, MM with RF appeared to have higher rates of transplant-related mortality (TRM) compared with the normal renal function (NRF) patients [11]. Although ASCT is still one of the disease's most effective treatments [12], the presence of coexistent renal disease limits the therapeutic options and stem cell transplant eligibility [13]. In recent years, several reports have shown that the use of ASCT is safe and effective in MM with RF [14–18]. However, there still have some considerable variabilities in reported survival outcomes and renal recovery from the limited literature, and the studies included have different priorities in clinical and renal response.

Herein, we fully summarized the studies of ASCT in MM with RF, including the prospective studies, the retrospective studies, and the case reports. The diagnosis, types, and mechanisms of RF in MM are also discussed. More importantly, we analyzed the data of renal recovery and clinical response to answer the question of clinical controversy following ASCT treatment and evaluate whether MM with RF benefits from ASCT or not.

## 2. Diagnosis, Types, and Mechanisms of RF in MM

### 2.1. Diagnosis of RF in MM before the ASCT Therapy

The classification guidelines for renal failure in MM were adapted in 2014 [19]; eGFR was used only in patients with stable renal function. From the studies we included, most of them were according to the novel International Myeloma Working Group (IMWG) criteria for symptomatic MM [19], and it is based on either reduced creatinine clearance (CrCl < 40 ml/min) or elevated serum creatinine (SCr > 2 mg/dl). Although the criteria are more sensitive for the determination and evaluation of renal failure in nephropathy, the standards of RF in our included studies are still inconsistent; the diagnosis of renal failure in MM requires relatively uniform standards in the future.

### 2.2. Types of RF in MM

MM-associated RF can be classified into the following different types: cast nephropathy (CN), light chain (LC) amyloidosis (AL), Fanconi syndrome, and monoclonal immunoglobulin deposition disease (MIDD). CN accounts for 33%, MIDD 22%, and light chain amyloidosis 21% [2]. MIDD includes LC deposition disease (LCDD), predominant deposits of kappa LC, heavy-chain deposition disease, and light heavy-chain deposition disease.

### 2.3. Mechanisms of RF in MM

Myeloma cast nephropathy is the major cause of renal failure in MM, which results from monoclonal LC precipitation with Tamm-Horsfall protein into casts that occlude the renal distal tubule lumens. Cast nephropathy develops when LC precipitation overcomes the capacity of tubular cells to catabolize and to endocytose the filtered free LCs [20, 21]. Moreover, nephrotoxic drugs (aminoglycoside antibiotics and nonsteroidal anti-inflammatory agents), hypercalcemia, dehydration, and contrast agents contribute to the development of renal failure [5, 22, 23]. As a result, the excess LCs form casts and aggregates with uromodulin in the distal nephron, leading to tubular obstruction and concomitant inflammation [20, 21, 24]. Furthermore, LC has direct toxic effects on kidney damage, and LC protein accumulates in renal tubular epithelial cells, inhibiting the metabolism of tubular cells and affecting the transportation of normal ions, amino acids, phosphates, etc. With the cast nephropathy developed, LCs can infiltrate the whole kidney and cause tubular, vascular, or glomerular damage. ASCT can effectively clear the LC, and renal damage may achieve remission; however, the data of renal function improvement are still limited.

## 3. ASCT in MM with RF: The Summarized Clinical Studies

There is growing concern about the curative effect of ASCT in MM with RF; more studies were reported to assess the clinical response and renal function in recent years. We fully summarized those studies but the included studies have different types, and the data of those studies were incomplete and variable. Therefore, we classified these studies into the cohort studies, the retrospective analysis studies, and the case reports, and the characteristics of each study are shown in Table 1. We fully summarized and classified the data of RF diagnosis, conditioning regimen, clinical response, survival, and response of renal function. Furthermore, we discovered that the present studies have different priorities in clinical and renal response; in the cohort studies, authors seemed to attach more weight to the clinical response. On the contrary, a retrospective analysis took more attention to renal function change. We also took a meta-analysis through the cohort study data to discuss whether the use of ASCT is safe and effective in MM with RF or not, and the data included the survival analysis, clinical response, and mortality.

### 3.1. ASCT in MM with RF: The Cohort Studies and Meta-Analysis

#### 3.1.1. Search Strategy

We performed a literature search in February 2019 in the Elsevier, EMBASE, Web of Science, and PubMed databases.

**Figure 1:**
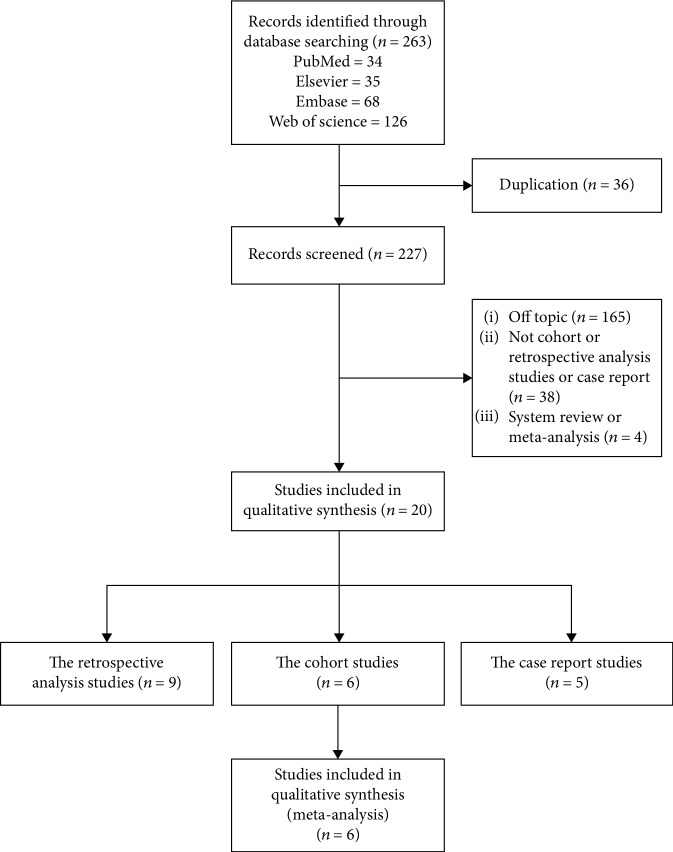
Flow diagram representing the selection process.

The following search terms were used: (1) “Autologous stem cell transplantation” or “Monoclonal Gammopathies” or “ASCT”; (2) “renal failure” or “renal function” or “acute kidney injury”; (3) “multiple myeloma” or “myeloma” or “MM”; and (4) “the cohort studies,” “the retrospective analysis studies,” and “the case report studies.” In addition, the reference lists of retrieved papers and recent reviews were reviewed. The flow diagram of search strategy is presented in Figure 1.

#### 3.1.2. Study Criteria

The inclusion criteria for studies were as follows: (1) the cohort studies comparing data on the clinical response and survival (“CR,” “PR,” “VGPR,” “OS,” “EFS,” “PFS,” and “TRM”); (2) validated diagnosis of renal failure and original research related to renal failure in MM patients; (3) studies that provided information about ASCT in MM with renal failure; and (4) articles that reported a clear comparison of RF (renal failure) population versus NRF (normal renal function) population controls with a direct effect on the clinical response and survival data.

The exclusion criteria were as follows: (1) duplicate studies; (2) studies such as systemic reviews, meta-analyses, and comments; and (3) studies of ASCT in MM with renal failure without detail research data in the clinical response and survival data.

#### 3.1.3. Data Extraction

Data extracted from each study included the first author's name, the publication year, the country of study origin, number of patients, median age, and the clinical response and survival (“CR,” “PR,” “VGPR,” “OS,” “EFS,” “PFS,” and “TRM”). If a study did not clearly mention any of the above key points, it had not performed the required methods. Two of the authors (Hongfei Zhong and Gaosi Xu) independently reviewed the selected studies and extracted data. Discrepancies were resolved by discussion.

#### 3.1.4. Statistical Analysis

The data was abstracted and analyzed using Stata (version 12) to make the outcomes more comprehensive. The binary variable outcomes were the EFS/PFE and OS; the data were expressed as the hazard ratio (HR) with 95% CI (confidence interval), and the estimation of the effect was performed by using a random effects model. Other binary variable outcomes were the PR and CR, and the date were expressed as the odds ratio (OR) with 95% CI (confidence interval); when combining studies, the random effects model was used to account for study heterogeneity. We used *Q* statistic and *I*^2^ tests to evaluate the heterogeneity. Low, moderate, and high heterogeneities were represented by thresholds of <25%, 25-75%, and >75%, respectively. *P* ≤ 0.05 was considered significant in all statistical tests.

#### 3.1.5. Data Analysis

**Figure 2:**
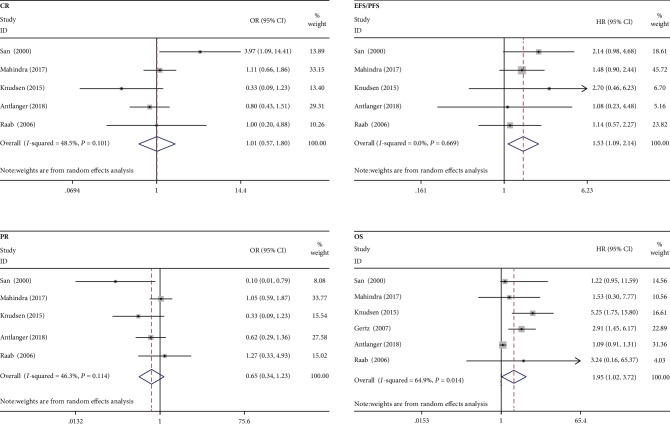
ASCT in myeloma patients with renal failure, survival analysis, and clinical response.

Recently, some studies reported the safety and clinical efficacy of ASCT use in myeloma patients with RF (Table 2) [16, 25–29]. Six articles [16, 25–29] with a total of 2930 MM patients were included in the meta-analysis. The binary variable outcomes were the incidence of overall survival (OS), event-free survival (EFS), progression-free survival (PFS), complete response (CR), partial response (PR), very good partial response (VGPR), and transplantation-related mortality (TRM). In addition, the data of OS and EFS expressed as the hazard ratio (HR) with 95% confidence interval (CI), and the data of CR, PR, VGPR, and TRM were expressed as the odds ratio (OR) with 95% CI; the estimation of the effect was performed by using a random effects model. The clinical response and survival analysis in MM with RF after ASCT are shown in Figure 2. To the best of our knowledge, this is the only and the first meta-analysis that reported the clinical response and survival data of ASCT treatment in MM with RF. Obviously, the results showed that the use of ASCT was associated with increased risk of mortality, and the outcome was consistent with the previous studies [13]. The CR (OR 1.013, 95% CI 0.569 to 1.804; *I*^2^ = 48.5%, *P* = 0.101) and PR (OR 1.013, 95% CI 0.342 to 1.226; *I*^2^ = 46.3%, *P* = 0.144) were not significantly different between the RF and NRF groups. Survival analysis indicated that MM with RF have lower survival rates (OS: HR 1.95, 95% CI 1.020 to 3.720; *I*^2^ = 64.9%, *P* = 0.014), and the major cause of a low survival rate in MM with RF may be due to the high toxicity in ASCT therapy. As a whole, ASCT was feasible and could lead to similar clinical response outcomes compared with those without advanced renal failure, but the survival analysis seemed to be not optimistic. Moreover, we noticed that the number of patients in some studies was relatively small. So large-size cohort studies are needed to prove this conclusion of ASCT for MM with RF in the future. Unfortunately, these reports had limit outcomes of renal response; only three studies [16, 26, 29] reported the renal function change.

### 3.2. ASCT in MM with RF: The Retrospective Analysis Studies

Nine retrospective analysis studies reported the outcome of ASCT treatment in MM with RF, these studies were done to mainly observe the alteration of the RF in MM patients. It was revealed that few studies focus on the clinical response and survival data and most retrospective studies tend to observe the renal response, and it was contrary to the emphasis of previous cohort studies [16, 25–29]. In general, fewer clinical response (CR, PR, and VGPR) was reported in the retrospective analysis studies. From the existing data, ASCT treatment seemed to have a better PR rate (62%), and the CR was 38% (Table 3). Augeul-Meunier et al. and Tosi et al. reported a good PR (96%, 67%); these studies mostly used low doses of melphalan [30, 31]. Badros et al. and Bernard et al. reported a good CR (50%, 43%), but the dose of melphalan was high [18, 32]. We indicated that the dose of melphalan escalation may result in higher response rates. Overall, from the retrospective studies, we conclude that ASCT as a good clinical response treatment could be an effective therapy in MM with RF.

Although cohort studies [16, 25–29] reported the clinical efficacy of ASCT use in MM with RF, however, the data of renal function response was less. We summarized the retrospective analysis studies that reported renal function response, and these studies complemented the renal response outcome of the previous cohort studies. Parikh et al. [33], Bernard et al. [18], Augeul-Meunier et al. [30], Ballester et al. [34], Balsam et al. [35], Glavey et al. [36], and Tosi et al. [31] reported the renal response after ASCT, and the improvements in renal function were 32%, 25%, 60%, 17%, 33%, 100%, and 83%, respectively. However, the definition of RF in each study was different. From the limited research, we found that lower-dose melphalan might have a better improvement of renal function (Augeul-Meunier et al. 60%, Glavey et al. 100%, and Tosi et al. 83%, respectively). On the contrary, the improvements of patients with renal recovery in the high-dose melphalan group were 32% and 25%. What is more, the USA Myeloma Group reported that the patients with RF underwent ASCT and ten patients (21%) experienced downstaging of renal failure [33]. It also reminds us that high doses of melphalan are associated with severe renal failure and should be used cautiously. On the other hand, age may also be an important factor affecting the curative effect of ASCT treatment. Tosi et al. [31, 37] reported a good renal function improvement, and the median ages were 49 and 47. A previous study also indicated that ASCT has been the mainstay of therapy in young patients with MM [10]. ASCT treatment may have age limitations, especially in patients with RF. However, some researches associated with older patients still have a safe and efficacy treatment of renal recovery [30, 36]; controversies exist about the benefits of transplantation for patients with older age. A future study needs to assess the effects of age values at the time of ASCT treatment in MM. Furthermore, patients in four retrospective analysis studies suffered a predialysis before ASCT [30, 33, 34, 36]. It appears from the data at hand that there is almost no connection between the predialysis and the outcome of ASCT therapy.

### 3.3. ASCT in MM with RF: The Case Report Studies

Five case report studies [37–41] were included in our research, and our summary is shown in Table 4. One patient reported an acute renal tubular necrosis, which may due to the consumption of cooked grass carp fish in the night. In contrast to those of other patients in the four studies, the renal functions were improved.

Two studies (Bigé et al. and Tauro et al.) have shown a renal improved advantage for patients who receive ASCT with a high-dose melphalan (200 mg/m^2^) treatment; this is in contrast to our retrospective study data. Historically, patients with RF either have received reduced doses or have been excluded from ASCT therapy with high-dose melphalan. Perhaps, the researchers prefer to report that high-dose melphalan may be safely administered to MM with RF. However, cohort studies with more patients are still necessary to assess the benefit of high-dose therapies in these cases.

## 4. Melphalan: Is It Safe for MM with RF?

Melphalan is probably the most effective chemotherapeutic agent in MM with a clear dose-response effect, and melphalan usually is a conditioning regimen before ASCT treatment. It has shown reduced overall mortality and improved PFS compared to conventional chemotherapy in MM [8, 9, 41, 42]. The standard conditioning regimen of melphalan (a dose of 200 mg/m^2^) was used for patients with NRF [43], melphalan has a dose-response antimyeloma effect, and higher doses could potentially improve the clinical response when used as a conditioning regimen for ASCT [44]. Unfortunately, melphalan has encountered dose-limiting toxicities, especially in MM with RF. Because of conflicting data on altered melphalan pharmacokinetics in renal insufficiency, patients with creatinine levels > 2 mg/dl have usually been excluded from high-dose melphalan treatment [45, 46]. However, some studies have found high-dose chemotherapy with melphalan can be administered to selected patients with RF [34, 40]. Our two case reports also come to the same conclusion [39, 41], and RF might no longer constitute a criterion for dose reduction or exclusion from such therapy.

In our summarized clinical studies, the data associated with melphalan dose were chaotic, and most studies showed that the dose of melphalan use was arbitrary (from 100 to 200 mg/m^2^), and the definition of high-dose melphalan was different in each study [30, 33]. In the cohort study groups, five researchers reported the use of melphalan as the conditioning regimen during the ASCT treatment [16, 25–28]; the dose of melphalan use may be the source of heterogeneity in the meta-analysis. Owing to the limited data of dose gradient of melphalan use, we cannot take a subgroup to assess whether the dose gradient of melphalan will affect the survival analysis of ASCT treatment in MM with RF. However, existing data concluded that remission rate may not be affected by the melphalan use (CR: OR 1.013, 95% CI 0.569 to 1.804; *I*^2^ = 48.5%, *P* = 0.101; PR: OR 1.013, 95% CI 0.342 to 1.226; *I*^2^ = 46.3%, *P* = 0.144), and the heterogeneity of data was acceptable. In the retrospective analysis studies, six studies used melphalan as the conditioning regimen, and we indicated that low-dose melphalan (melphalan 80 mg/m^2^, 140 mg/m^2^) treatment might have a lower mortality [30, 32], but with the increase of melphalan doses, the TRM was increased [18]. Furthermore, low doses of melphalan use may achieve a good PR [30, 31], and high doses might have a good benefit in CR [18, 32]; the dose of melphalan escalation may result in higher response rates. We also found low-dose melphalan (melphalan 80 and 140 mg/m^2^) treatment might have a lower mortality [30, 32], but with increasing doses of melphalan, the data of survival analysis was controversial. As for the renal improvement aspect, low-dose melphalan use has demonstrated a good renal recovery from the retrospective studies. However, with the process of the increased dose, changes in renal function have been described in different outcomes, so clinical trials are required for more evaluation of high-dose melphalan use in MM with RF, especially in renal recovery outcomes.

## 5. Conclusion

Accumulating evidence suggests that in MM with RF, ASCT could be a feasible therapy and can lead to similar remission outcomes to those without advanced RF. Our current study indicated that the MM with RF after ASCT truly has a good improvement of renal function but has a low survival rate. For the recovery of kidney function in MM patients, ASCT may probably be a friend, but it may be a foe due to the low survival rate. In general, from the overall efficacy, ASCT is worth a try in MM patients with RF. The clinical response of the conditioning melphalan therapy in RF patients remains controversial, especially in dose response of melphalan use. Moreover, melphalan is still an important factor affecting the treatment of ASCT.

## Figures and Tables

**Table 1:** ASCT in myeloma patients with renal insufficiency, the characteristics of the studies.

Author	Year	Country	Diagnosis of RF	Renal failure clinical stage in MM patient	Dialysis or not before ASCT	Therapy	Prognostic criteria
ASCT in myeloma patients with renal insufficiency: the cohort studies							
Antlanger et al. [29]	2018	Austria	eGFR < 60 ml/min (MDRD)	ISS stage I (14%) II (30%) III (54%)	Not	Conventional chemotherapy+ASCT	eGFR (MDRD)
Gertz et al. [28]	2007	USA	SCr > 2 mg/dl	ISS stage I (0%) II (20%) III (80%)	Not	Conditioning regimen (Mel)+ASCT	NR
Knudsen et al. [16]	2015	Denmark	CrCl < 60 ml/min	ISS stage II (8%) III (21%)	NR	Conditioning regimen (Mel)+ASCT	NR
Mahindra et al. [27]	2017	USA	eGFR < 30 ml/min (MDRD)	Severe RF	Not	Conditioning regimen (Mel)+ASCT	NR
Raab et al. [25]	2006	USA	SCr > 2 mg/dl	ISS stage I (14%) II (10%) III (74%)	NR	Conditioning regimen (Mel)+ASCT	NR
San Miguel et al. [26]	2000	Spanish	SCr > 2 mg/dl	ISS stage II (14%) III (86%)	Not	Conditioning regimen (Mel)+ASCT	CrCl

ASCT in myeloma patients with renal insufficiency: the retrospective analysis studies							
Badros et al. [32]	2001	USA	Creatinine > 176.8 *μ*mol/l	NR	Not	Conditioning regimen (Mel)+ASCT	NR
Augeul-Meunier et al. [30]	2018	France	CrCl < 30 ml/min	NR	Dialysis dependence (47%)	Conditioning regimen (Mel)+ASCT	NR
Ballester et al. [34]	1997	USA	SCr > 3 mg/dl	NR	Dialysis dependence (67%)	BUCY+ASCT	SCr
Balsam et al. [35]	2017	USA	GFR	CKD stage Stage 1 (31.8%) Stage 2 (43.8%) Stage 3 (17.7%) Stage 4 (3.1%) Stage 5 (1.6%)	Not	Conventional chemotherapy+ASCT	GFR
Bernard et al. [18]	2015	Canada	NR	NR	Not	Conditioning regimen (Mel)+ASCT	NR
Glavey et al. [36]	2011	USA	SCr > 3 mg/dl	ISS stage I (14%) II (10%) III (74%)	Dialysis dependence (53%)	Conditioning regimen (Mel)+ASCT	CrCl
Seok Hui et al. [47]	2011	Korea	eGFR (MDRD)	CKD stage IIIa (78%) IIIb (12%)	Not	Conditioning regimen (Mel)+ASCT	eGFR (MDRD)
Parikh et al. [33]	2009	USA	SCr > 2 mg/dl	NR	Dialysis dependence (20%)	Conditioning regimen (Mel)+ASCT	eGFR (MDRD)
Tosi et al. [31]	2000	Italy	CrCl < 40 ml/h	CKD stage IIIb (100%)	Not	Conventional chemotherapy+ASCT	CrCl

ASCT in myeloma patients with renal insufficiency: the case report studies							
Bigé et al. [39]	2009	France	SCr	Acute renal failure	Not	Conditioning regimen (Mel)+ASCT	SCr
Lam et al. [38]	2004	China	Normal renal function	Normal renal function	Not	ASCT	NR
Rebibou et al. [40]	1997	France	NR	Severe renal failure	Not	Conditioning regimen (Mel)+ASCT	NR
Reiter et al. [37]	1999	Austria	NR	NR	Not	Conditioning regimen (VAD)+ASCT	CrCl
Tauro et al. [41]	2002	UK	NR	NR	Not	Conditioning regimen (Mel)+ASCT	SCr

ASCT: autologous stem cell transplantation; RF: renal failure; CrCl: creatinine clearance; SCr: serum creatinine; NR: not reported; MDRD: Modification of Diet in Renal Disease; eGFR: estimated glomerular filtration rate; ISS: international staging system; CKD: chronic kidney diseases; BUCY: Busulfan and Toxicity cyclophosphamide; Mel: melphalan; GFR: glomerular filtration rate; VAD: dexamethasone.

**Table 2:** ASCT in myeloma patients with renal failure, the cohort studies.

Author	Country	No.	Median age	Diagnosis of RF	Conditioning regimen	NRF/RF	Clinical response and survival (NRF/RF) (%)							Response of renal function in RF group
							CR	PR	VGPR	OS	EFS	PFS	TRM	
Antlanger et al. [29]	Austria	288	57	eGFR < 60 ml/min (MDRD)	Conventional chemotherapy	238/50	41/36	26/17	28/28	70/68	NR	29/27	NR	Creatinine 2.6 mg/ml decreased to 2.0 mg/ml and eGFR 33 increased to 41 ml/min/1.73 m^2^
Gertz et al. [28]	USA	677	59	SCr > 2 mg/dl	Melphalan (140/200 mg/m^2^)	637/40	NR	NR	NR	48/24	NR	NR	NR	NR
Knudsen et al. [16]	Denmark	107	56	9	Melphalan (100/140/200 mg/m^2^)	78/29	93/83	93/83	NR	85/52	50/27	NR	1/17	10 patients reached a normal renal function
Mahindra et al. [27]	USA	1307	60	eGFR < 30 ml/min (MDRD)	Melphalan (140/200 mg/m^2^)	1240/67	32/34	23/24	30/16	70/60	NR	35/27	25/33	NR
Raab et al. [25]	USA	34	58	SCr > 2 mg/dl	Melphalan (100/200 mg/m^2^)	17/17	53/59	24/24	NR	70/42	20/18	NR	6/6	NR
San Miguel et al. [26]	Spanish	493	55	SCr > 2 mg/dl	Melphalan (140 mg/m^2^)	479/14	48/80	43/10	NR	61/56	NR	44/27	3.3/29	6 patients reached levels of creatinine 2 mg/dl and CrCl 50 ml/min

ASCT: autologous stem cell transplantation; RF: renal failure; CrCl: creatinine clearance; SCr: serum creatinine; OS: overall survival; EFS: event-free survival; PFS: processing free survival; CR: complete response; PR: partial response; VGPR: very good partial response; TRM: transplantation related mortality; NR: not reported; MDRD: Modification of Diet in Renal Disease; eGFR: estimated glomerular filtration rate.

**Table 3:** ASCT in myeloma patients with renal insufficiency: the retrospective analysis studies.

Author	Country	No.	Median age	Diagnosis of RF	Conditioning regimen	Clinical response and survival (%)							Response of renal function in the RF group
						CR	PR	VGPR	OS	EFS	PFS	TRM	
Badros et al. [32]	USA	81	53	Creatinine > 176.8 *μ*mol/l	Mel140 (26%) Mel200 (74%)	58	NR	NR	55	48	NR	6	NR
Augeul-Meunier et al. [30]	France	55	61	CrCl < 30 ml/min	Mel140 (87%) Mel200 (13%)	43	96	58	72	NR	45	6	10 patients (18%) presented minor renal response and 1 with partial renal response Proteinuria decreased for the majority of patients (60%)
Ballester et al. [34]	USA	6	50	SCr > 3 mg/dl	BUCY	17	50	NR	50	NR	NR	50	1 patient (17%) has shown a progressive recovery of renal function (SCr was decreased)
Balsam et al. [35]	USA	192	57.1	GFR	Conventional chemotherapy	NR	NR	NR	NR	NR	NR	NR	64 patients (33%) reversed renal failure (GFR was increased)
Bernard et al. [18]	Canada	33	56	NR	Mel 140 (36%) Mel 160 (3%) Mel 200 (61%)	50	46	50	63	NR	NR	15	7 patients (25%) had an improved renal function
Glavey et al. [36]	USA	30	61	SCr > 3 mg/dl	NR	NR	NR	NR	NR	NR	NR	NR	Average creatinine 4.9 mg/dl decreased to 3.9 mg/dl
Seuk Hui et al. [47]	Korea	41	49	eGFR (MDRD)	Mel 100 (100%)	NR	NR	NR	NR	NR	NR	NR	Average eGFR decreased in 24 months
Parikh et al. [33]	USA	46	56	SCr > 2 mg/dl	Mel 140 (6%) Mel 180 (29%) Mel 200 (65%)	22	53	NR	64	NR	36	NR	15 patients (32%) experienced a sustained improvement in renal function (eGFR was increased) 10 patients (21%) experienced a downstaging of renal failure
Tosi et al. [31]	Italy	6	47	CrCl < 40 ml/h	Mel 80 (83%)	0	67	0	NR	NR	NR	NR	5 patient (83%) have shown increased CrCl

ASCT: autologous stem cell transplantation; BUCY: Busulfan and Toxicity cyclophosphamide; RF: renal failure; Mel: melphalan; CrCl: creatinine clearance; SCr: serum creatinine; GFR: glomerular filtration rate; eGFR: estimated glomerular filtration rate; OS: overall survival; EFS: event-free survival; PFS: processing free survival; CR: complete response; PR: partial response; VGPR: very good partial response; TRM: transplantation related mortality; NR: not reported; MDRD: Modification of Diet in Renal Disease.

**Table 4:** ASCT in myeloma patients with renal insufficiency: the case report studies.

Author	Year	Country	Age	Immunochemical subtype	Renal function before ASCT	Treatment	Clinical response and renal function after ASCT
Bigé et al. [39]	2009	France	57/56	Case 1: light chain Case 2: IgA	Case 1: acute renal failure, SCr 673 *μ*mol/l Case 2: acute renal failure, SCr 576 *μ*mol/l	Case1: ASCT was performed after high-dose melphalan (200 mg/m^2^) Case 2: treated with five courses of VAD chemotherapy and then received ASCT	Case 1: SCr 673 *μ*mol/l decreased to 280 *μ*mol/l Case 2: SCr 576 *μ*mol/l decreased to 450 *μ*mol/l
Lam et al. [38]	2004	China	63	IgA	Normal renal function	Received a non-myeloablative ASCT	Acute renal tubular necrosis
Rebibou et al. [40]	1997	France	49	IgG	Severe renal failure	The therapeutic regimen consisting of one high-dose melphalan infusion and ASCT was infused 5 days after melphalan	CR: 14 months Renal function: NR
Reiter et al. [37]	1999	Austria	51	Light chain	SCr 1.9 mg/dl	Conventional therapy with VAD, then ASCT infused	CrCl had improved to 46 ml/min CR: 1 year
Tauro et al. [41]	2002	UK	52	NR	SCr 690 *μ*mol/l	The patient was treated with high-dose melphalan (200 mg/m^2^); then ASCT was infused	SCr 690 *μ*mol/l decreased to 429 *μ*mol/l

ASCT: autologous stem cell transplantation; MM: multiple myeloma; SCr: serum creatinine; CrCl: creatinine clearance; CR: complete response; VAD: vincristine, adriamycin, and dexamethasone; NR: not reported.

## Abbreviations

RF: Renal failure
MM: Multiple myeloma
ASCT: Autologous stem cell transplantation
NRF: Normal renal function
AKI: Acute kidney injury
LC: Light chain
MIDD: Monoclonal immunoglobulin deposition disease
LCDD: LC deposition disease
CN: Cast nephropathy
IL: Interleukin
HR: Hazard ratio
OR: Odds ratio
RIFLE: Risk, Injury, Failure, Loss and End-Stage Kidney Disease
AKIN: Acute Kidney Injury Network
IMWG: International Myeloma Working Group
MDRD: Modification of Diet in Renal Disease
CrCl: Creatinine clearance
SCr: Serum creatinine
OS: Overall survival
EFS: Event-free survival
PFS: Progression-free survival
CR: Complete response
PR: Partial response
VGPR: Very good partial response
TRM: Transplantation-related mortality.

## Additional Points

*Highlights.* (i) The studies of ASCT in MM with RF were fully summarized. (ii) The diagnosis, types, and mechanisms of RF in MM were discussed. (iii) The data of renal recovery and clinical response during ASCT treatment were analyzed. (iv) Whether MM with RF benefits from ASCT or not was evaluated. (v) Melphalan is still an important factor affecting the treatment of ASCT.
